# HQA-Data: A historical question answer generation dataset from previous multi perspective conversation

**DOI:** 10.1016/j.dib.2023.109245

**Published:** 2023-05-18

**Authors:** Sabbir Hosen, Jannatul Ferdous Eva, Ayman Hasib, Aloke Kumar Saha, M.F. Mridha, Anwar Hussen Wadud

**Affiliations:** a Department of Computer Science and Engineering, University of Asia Pacific, Dhaka, Bangladesh; b Department of Computer Science, American International University-Bangladesh, Dhaka, Bangladesh; c Department of Computer Science and Engineering, Bangladesh University of Business and Technology, Dhaka, Bangladesh

**Keywords:** Natural language processing, Deep learning, BERT, Machine learning, Question answering generation, Ubuntu dialogue corpus, Text processing

## Abstract

This data article contains a quality assurance dataset for training the chatbot and chat analysis model. This dataset focuses on NLP tasks, as a model that serves and delivers a satisfactory response to a user's query. We obtained data from a well- known dataset known as “The Ubuntu Dialogue Corpus” for the purpose of constructing our dataset. Which consists of about one million multi-turn conversations containing around seven million utterances and one hundred million words. We derived a context for each dialogueID from these lengthy Ubuntu Dialogue Corpus conversations. We have generated a number of questions and answers based on these contexts. All of these questions and answers are contained within the context. This dataset includes 9364 contexts, 36,438 question-answer pairs. In addition to academic research, the dataset may be used for activities such as constructing this QA for another language, deep learning, language interpretation, reading comprehension, and open-domain question answering. We present the data in raw format; it has been open sourced and publicly available at https://data.mendeley.com/datasets/p85z3v45xk.

**Specifications Table**

**Table:** 

Subject:	Human-Computer Interaction
Specific subject area:	A question answering dataset that can be useful for historical chat analysis which can also be used for QA from users previous conversation.
Type of data:	Text
How the data were acquired:	We acquire the chatlogs from the Ubuntu Dialogue Corpus dataset. And from this dataset based on dialogueID, we transformed the conversations in contexts. From the context we generate Question Answer pairs.
Data format:	Secondary data
Description of data collection:	Convert the raw human dialogues from the Ubuntu Dialogue Corpus into a context. Generate various questions and responses from each context.
Data source location:	The Ubuntu Dialogue Corpus: A Large Dataset for Research in Unstructured Multi-Turn Dialogue Systems Paper: https://arxiv.org/abs/1506.08909 Dataset: https://www.kaggle.com/datasets/rtatman/ubuntu-dialogue-corpus
Data accessibility:	Repository name: HQA-data: A historical Question Answer Generation dataset From previous multi perspective conversation Data identification number: 10.17632/p85z3v45xk.1 Direct URL to data: https://data.mendeley.com/datasets/p85z3v45xk

## Value of the Data

• This is a dataset for question answering that is created using the chat logs or conversation histories of users. It is unique because it is the only dataset that uses user dialogues.
• The dataset is a valuable resource for training and testing chatbots and chat analysis algorithms in NLP research and related fields, as well as for improving the performance of conversational AI systems in businesses.
• The dataset can also be used by students to learn and practice NLP approaches, and by anyone interested in NLP and AI to explore various techniques.
• By modifying this dataset, it can be utilized for the development of language models in other languages, such as 'Bengali,' as well as in other domains.
• This dataset also can be used in Reading Comprehension tasks. To understand the contexts and find the answers based on questions.

## 1 Objective

Our dataset is derived from a popular dataset, The Ubuntu Dialogue Corpus is composed of about one million talks extracted from the Ubuntu chat logs, which were used to acquire technical help for a variety of Ubuntu-related issues [1]. This is an established dataset containing the chat history of users, but no QA-formatted dataset was available for this data. Therefore, we have built a whole new Question Answering dataset. Where we have transformed the user chat log to the context and generated questions and answers based on the conversation history.

## 2 Data Description

A chatbot is a robot that responds with relevant responses to customer questions. Chat analysis is the process of evaluating a conversation or collecting relevant information, as well as monitoring the behaviour or sentiment of users. A valuable question-response dataset is required for the development of a chatbot and a chat analysis model. In light of this, we have derived a QA dataset from a primary dataset, The Ubuntu Dialogue Corpus. This Ubuntu Dialogue Corpus dataset only covers multi-turn conversations between people [1]. We manually read out the QA pairs from the generated Question Answers, and if the QA is relatable then we kept that QA pair. It helps to increase the overall precision of the QA system. It is available to the public to help and encourage more research into making automated QA.

Our created dataset is available in a data repository at mendeley data and there is a folder called Dataset. In this Dataset folder there are four different files. These four files are test_data.csv, test_data_json_file.json, train_data.csv, train_data_json_file.json.

Fig. 1

**Fig. 1:**
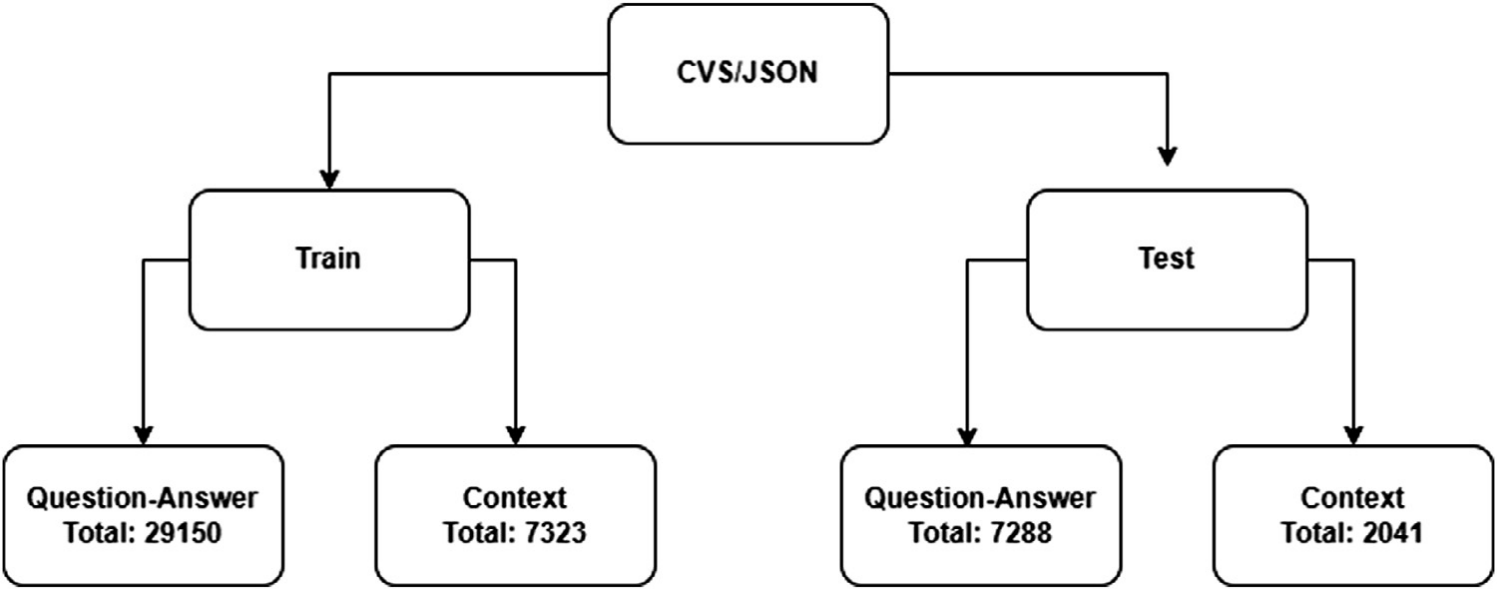
CSV/JSON formatted data structure.

Our dataset is shown to be represented in two different data formats, the first of which is data formatted in CSV, and the second of which is data formatted in JSON, as seen in figure-01. The Train section of each of these files has a total of 29,150 question-answer pairs and a total of 7323 context pairs. The Test section files contain the question-answer pairs and context. There are a total of 7288 question-answer pairs and a total of 2041 contexts in the Test section Table 1.

**Table 1:** Name of the columns with the descriptions of the CSV files.

Column Name	Description
dialogueID	The string representation of the unique identifier for each context.
Context	The text representation, which contains the whole conversation.
QuestionID	The string representation of the unique identifier for each generated question.
Question	The representation of the string which contains questions generated from the context.
Answer	Contains the answers to the generated questions. This is also the representation of the strings.
Answer Start	This is an integer number that contains the starting index of the answer from the context.
Answer End	This is an integer number that contains the ending index of the answer from the context.

The names of the columns with brief explanations of their contents of the CSV files of the dataset are found in table-01. The names of these columns are dialogueID, which is the unique identifier of the conversation, Context, which contains the overall conversation that has been contributed by multiple users, QuestionID, which is the unique identifier for both Questions and Answers, Answer start, and Answer end, which are the starting and ending indexes of the Answer for the Question.

**Fig. 2:**
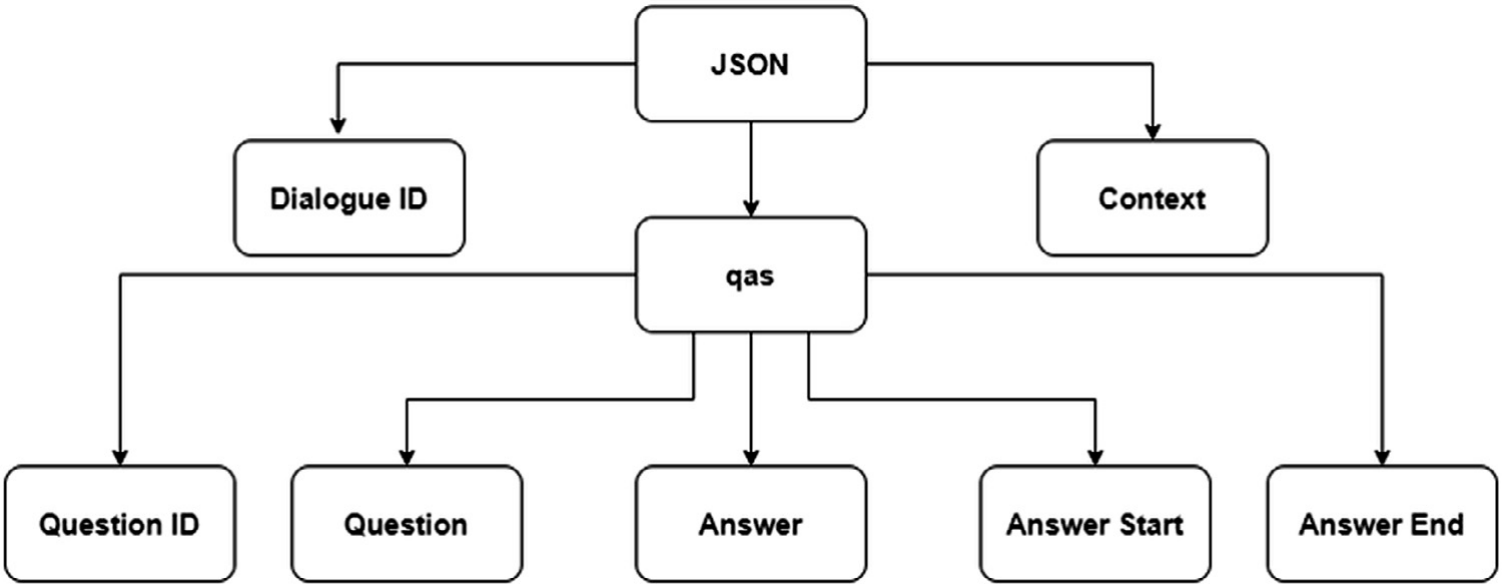
JSON formatted data structure.

In our dataset Fig. 2, is showing the structural representation of JSON files. It is containing the following attributes: dialogueID which is unique of each context, Context is containing the users conversation, qas which is a list of objects and the elements of that objects are QuestionID, Question, Answer, Answer Start, and Answer End.

## 3 Experimental Design, Materials and Methods

**Fig. 3:**
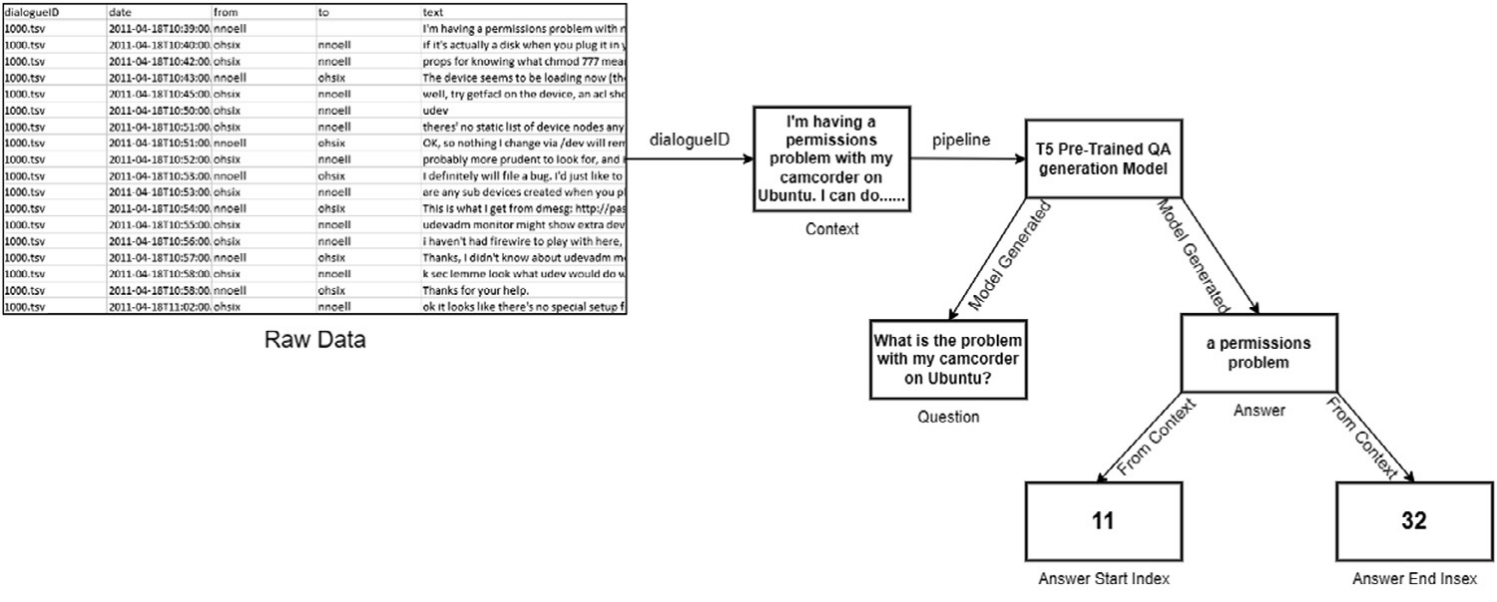
Data creation process.

The source of the raw data that we collect is the "Ubuntu Dialogue Corpus," which consists of almost one million conversations between more than one person extracted from the Ubuntu chat logs and used to receive technical support for various Ubuntu-related problems [1]. We get those data in csv files that have five columns dialogueID, date, from, to and text. ‘dialogueID’ is the unique conversation id on a related topic. Each chat room, or channel, has a particular topic, and we can separate those topics using dialogueID that is in csv. Using dialogueID we can extract chat logs in a context that will be context of chat logs. In the corpus there are numerous contexts that are very large that are more than approximately 10,000 words. To read all the context and generate is more difficult and more complex to do. We are using a question answer generator T5 model [3]. This pre-trained model generates questions and their accompanying replies depending on the given context or passage. Our extracted context from the raw dataset that passes to the T5 model will generate qa pairs. It generates more than 9 lakh question answer pairs. Though it is a model not well pre trained in that it generates more garbage and unrelated questions. So, we read the question answer pairs and if it has meaning and is related to our context, we keep only those pairs. We are able to get  only 36,438 question-answer pairs from 9363 contexts. We are following the format of SQuAD V2 reading comprehension dataset [2]. In our dataset we have context, question, question, answer, and the starting and ending position of answer in the context. In the Fig. 3 we can see a process of how we generate qa pairs.

### 3.1 Efficiency Test of this Dataset with some Algorithms

We are testing our datasets in Hugging Face transformers pre-trained model fine tuning using pytorch with two different models Bidirectional Encoder Representations from Transformers (BERT) [4]. And second one is DistilBERT is a small, fast, cheap and light Transformer model trained by distilling BERT base [5]. With a wrapper of question answering that model can extract the question answer from the given context. To measure the model, we are using different metrics: Exact match, F1 score, Rouge, and BLEU. Table-02 shows the score of the models.

Our dataset is trained using two different transformer-based models derived from Hugging Face. The metrics that they use for evaluation are presented in Table 2 below. Both models are trained on up to three epochs, as shown in the table; nevertheless, the BERT model achieves the most exact match percentage of 60% and the highest F1-score percentage of 70.73%.

**Table 2:** Evaluation Metrics of BERT and DistilBERT.

Model	Epoch	Exact Match	F1 Score	Rouge1	Rouge2	RougeL	RougeLSum	BLEU
**BERT**	1	56	66.57	66.78	20	66.65	66.82	22.8
	2	60	71.25	71.79	19.8	71.67	71.89	20.6
	3	60	70.73	71.33	23	71.07	71.33	37.95
**DistilBERT**	1	48	57.96	57.65	19	57.73	57.7	19.54
	2	54	64.07	63.93	18.6	63.84	65.27	22.02
	3	60	68.6	68.67	19.8	69.14	69.14	39.51

## CRediT authorship contribution statement

**Sabbir Hosen:** Software, Validation, Data curation, Investigation, Writing – original draft, Visualization. **Jannatul Ferdous Eva:** Validation, Data curation, Investigation, Writing – original draft, Visualization. **Ayman Hasib:** Validation, Data curation, Investigation, Writing – original draft, Visualization. **Aloke Kumar Saha:** Supervision, Writing – review & editing, Project administration. **M.F. Mridha:** Supervision, Writing – review & editing, Project administration. **Anwar Hussen Wadud:** Writing – review & editing.

## Declaration of Competing Interests

The authors declare that they have no known competing financial interests or personal relationships that could have appeared to influence the work reported in this paper.

## Data Availability

HQA-Data: A Historical Question Answer Generation Dataset from Previous Multi Perspective Conversation (Original data) (Mendeley Data).

## References

[1] Lowe, R., Pow, N., Serban, I., & Pineau, J. (2015). The ubuntu dialogue corpus: a large dataset for research in unstructured multi-turn dialogue systems. arXiv preprint.

[2] Rajpurkar, P., Zhang, J., Lopyrev, K., & Liang, P. (2016). Squad: 100,000+ questions for machine comprehension of text. arXiv preprint.

[3] Patil, S. Question Generation using transformers (Version 1.0.0) [Computer software]. https://www.github.com/patil-suraj/question_generation.

[4] Devlin, J., Chang, M.W., Lee, K., & Toutanova, K. (2018). Bert: pre-training of deep bidirectional transformers for language understanding. arXiv preprint.

[5] Sanh, V., Debut, L., Chaumond, J., & Wolf, T. (2019). DistilBERT, a distilled version of BERT: smaller, faster, cheaper and lighter. arXiv preprint.

